# Examining Sex Differences in Conditioned Place Preference or Aversion to Methamphetamine in Adolescent and Adult Mice

**DOI:** 10.3389/fphar.2021.770614

**Published:** 2021-11-30

**Authors:** Ellen R. Cullity, Alexandre A. Guerin, Christina J. Perry, Jee Hyun Kim

**Affiliations:** ^1^ Mental Health Theme, The Florey Institute of Neuroscience and Mental Health, Parkville, VIC, Australia; ^2^ Florey Department of Neuroscience and Mental Health, University of Melbourne, Parkville, VIC, Australia; ^3^ School of Medicine, IMPACT—The Institute for Mental and Physical Health and Clinical Translation, Deakin University, Geelong, VIC, Australia

**Keywords:** adolescence, methamphetamine, conditioned place preference, conditioned place aversion, sex differences

## Abstract

Adolescence marks a particularly vulnerable period to developing substance use disorders. Human and rodent studies suggest that hypersensitivity to reward may contribute towards such vulnerability when adolescents are exposed to casual drug use. Methamphetamine is a popular illicit substance used by male and female youths. However, age- and sex-specific research in methamphetamine is scarce. The present study therefore aimed to examine potential sex differences in methamphetamine-conditioned place preference in adolescent and adult mice. Mice (*n* = 16–24/group) were conditioned to methamphetamine (0.1 mg/kg). We observed that regardless of age, females were more hyperactive compared to males. Individually normalized score against baseline preference indicated that on average, adolescents formed stronger preference compared to adults in both sexes. This suggests that adolescents are more sensitive to the rewarding effects of methamphetamine compared to adults. Surprisingly, individual data showed that some mice formed a conditioned place aversion instead of preference, with females less likely to form an aversion compared to males. These results suggest that adolescents may be hypersensitive to methamphetamine’s rewarding effects. In addition, female resistance to the aversive effects of methamphetamine may relate to the sex-specific findings in humans, including quicker transition to regular methamphetamine use observed in females compared to males.

## Introduction

Methamphetamine is the most widely used illicit substance globally other than cannabis (United Nations Office on Drugs and Crime, 2017a). Youth use is particularly high (Degenhardt et al., 2016; United Nations Office on Drugs and Crime, 2017b; Australian Institute of Health and Welfare, 2020), which is a significant public and social health concern. A major factor in transition from casual to compulsive substance use is the early age of onset of drug use (Anthony and Petronis, 1995). Despite this, research on methamphetamine use in adolescence has lagged behind that of older ages and other substances (Luikinga et al., 2018). Especially lacking is information on how the biological sex may affect methamphetamine-related behaviors in adolescence. Unlike other illicit substances, prevalence of methamphetamine use is often similar in males and females across different countries (Compton et al., 2007; Courtney and Ray, 2014; Australian Institute of Health and Welfare, 2017; Papamihali et al., 2021). In addition, females transition more rapidly from initial to problematic methamphetamine use and are more sensitive to its acute subjective and behavioral effects compared to males (Brecht et al., 2004; Mayo et al., 2019).

Human and rodent studies suggest that hypersensitivity to reward contributes towards susceptibility to develop substance use disorder when exposed to casual drug use in adolescence (Zakharova et al., 2009; van Duijvenvoorde et al., 2016; Marshall et al., 2017). Conditioned place preference (CPP) is a widely used rodent paradigm that evaluates whether a substance is experienced as rewarding. Typically, in CPP, rodents learn to associate a distinct environment (context) with a drug experience. This is achieved by repeatedly pairing drug administration with one distinct chamber while another distinct chamber is paired with vehicle or saline. On test day, animals are given unrestricted access to both chambers without any drug administration. If the rodent spends more time in the drug-paired compared to the saline-paired chamber, it suggests that the rodent prefers to spend time where the drug was given because the drug experience was rewarding. CPP thus is used as a direct measure of a drug's rewarding properties. Comparable CPP between adolescent and adult rats has been reported with 0.125–2 mg/kg methamphetamine, although only males were examined (Zakharova et al., 2009; Kim et al., 2013). Observations in adults indicate male and female rats are equally sensitive to forming a CPP to 0.1 mg/kg methamphetamine (Schindler et al., 2002), while female mice form more CPP to 1 mg/kg methamphetamine compared to males (Chen et al., 2003). Notably, none of those previous studies examined adolescents and adults of both sexes. Further, individual rodent CPP score was not conveyed to assess the likelihood of each age or sex forming a place preference. A recent study examined adolescent and adult mice of both sexes in methamphetamine-induced CPP (Cullity et al., 2021). It demonstrated that 3 mg/kg of methamphetamine is not more rewarding or less aversive in adolescents compared to adult mice, and that female mice are less likely than males to form CPP or conditioned place aversion (CPA) at this dose. That study used the highest dose reported for a methamphetamine-induced CPP study in mice to study potential age and sex differences in the aversive properties of methamphetamine (Cullity et al., 2021). Whether adolescents, especially females, show hypersensitivity to methamphetamine's rewarding effects measured by CPP to a low dose of methamphetamine is yet unknown.

The aim of this study is to examine potential age and sex differences in CPP to 0.1 mg/kg methamphetamine in mice. We also investigated methamphetamine-induced locomotion and conditioned hyperactivity. Methamphetamine injected at 0.1 mg/kg was chosen because it is the lowest dose shown to produce CPP in adult rodents (Schindler et al., 2002). Consistent with the model of adolescent hypersensitivity to reward (Spear, 2000; Casey et al., 2008; Steinberg et al., 2009; Zakharova et al., 2009; Friemel et al., 2010; Steinberg, 2010; Doremus-Fitzwater and Spear, 2016; van Duijvenvoorde et al., 2016; Marshall et al., 2017), we hypothesized that more adolescent mice would form a preference to this low dose of methamphetamine than adult mice. Based on Schindler et al. (2002), sex differences in adults are not expected for CPP, but females may be more sensitive to methamphetamine-induced hyperlocomotion (Schindler et al., 2002). In humans, females transition more rapidly from initial to problematic methamphetamine use (Brecht et al., 2004; Mayo et al., 2019); therefore, adolescent female mice may show the most CPP.

## Materials and Methods

### Animals

A total of 166 mice bred at The Florey Institute of Neuroscience and Mental Health were used in this study. The methods of this study are largely identical to a previous study (Cullity et al., 2021), except that a new set of naïve mice were used to acquire CPP with a ×30 lower dose of methamphetamine. Mice were originally generated by the Gene Expression Nervous System Atlas (GENSAT) program at the Rockefeller University, NY, United States (Gong et al., 2003), and bred on an Outbred Arc:Arc(S) Swiss background. On day 1 of experimentation, mice were either postnatal day (P) 49 ± 2 [referred to as adolescent throughout this paper; see Cullity et al. (2021)] or P70 ± 4 (referred to as adult throughout this paper). In open-top cages (34 cm × 16 cm ×16 cm), mice were housed with three to five littermates in same-sex groups and were maintained on a 12 h light/dark cycle (lights on at 07:00) at 22 ± 1.5°C. Water and food (standard chow: Barastoc, VIC, Australia) were available *ad libitum*. All procedures were approved by the Animal Care and Ethics Committee at the Florey Institute of Neuroscience and Mental Health in accordance with The Australian Code of Practice for the Care and Use of Animals for Scientific Purposes (NHMRC, 2013). Our study was sufficiently powered, with the initial target group size (total N = 144) calculated using G*Power (Faul et al., 2007): type 1 and 2 error rate = 0.05, small to medium effect size (Cohen’s f = 0.2), and repeated-measures design with 8 groups (2 ages × 2 sexes × 2 drugs due to saline only control).

### Methamphetamine

Methamphetamine (Sigma-Aldrich Australia Pty Ltd., NSW, Australia) was dissolved in 0.9% saline and intraperitoneally (i.p.) injected at 0.1 mg/kg per injection. This dose was chosen as it is the lowest dose previously shown to produce CPP in adult rodents (Schindler et al., 2002). All methamphetamine or saline injections were at 10 ml/kg volume.

### Conditioned Place Preference

The CPP protocol of this study followed a previous study (Cullity et al., 2021). Briefly, the experimental apparatus (Lafayette Instruments, IN, United States) comprised two main compartments with differences in visual (wall patterns) and tactile (floor texture) cues, separated by a neutral compartment. The light intensity settings were set at 30 (80 lux) within the conditioning compartments and 90 (380 lux) in the central compartment. Horizontal optic sensor beams and apparatus software (Motor MonitorTM, Kinder Scientific, United States) recorded the distance travelled and the time spent in each compartment.

**FIGURE 1:**
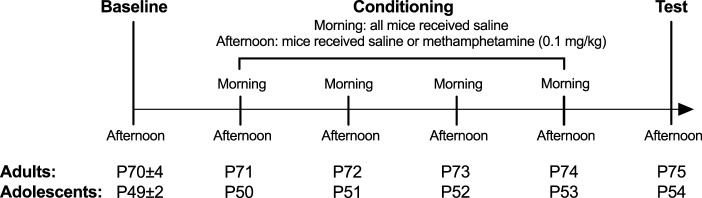
Experimental timeline. Sample size for saline groups: adolescent male *n* = 23; adolescent female: *n* = 18; adult male: *n* = 24; adult female *n* = 18 and for methamphetamine groups: adolescent male *n* = 22; adolescent female: *n* = 20; adult male: *n* = 21; adult female *n* = 20.

The CPP protocol and timeline are as described previously (Faul et al., 2007; Cullity et al., 2021) (Figure 1). In brief, mice were placed in the central compartment and allowed free access to all three compartments on day 1 afternoon (Baseline). On days 2–5 mornings (08:30–11:30), mice were i.p. injected with saline and were immediately confined within one of the compartments. On days 2–5 afternoons (12:00–15:00), mice received either an i.p. injection of methamphetamine (0.1 mg/kg) or saline and were immediately confined into the other compartment (Conditioning). We used an unbiased CPP protocol. That is, the afternoon compartment (in which control mice receive saline and CPP mice receive methamphetamine) was randomly allocated without an expectation of CPP or CPA. The other compartment served as the morning saline chamber control. Chamber allocation was counterbalanced across every condition. The test occurred on day 6 afternoon, with mice given free access to all three compartments without any injection. Each session occurred at the same time each day for a given mouse and was 30 min long.

CPP scores were calculated as reported previously (preference % = time in afternoon chamber/total time in both chambers x 100) (Cullity et al., 2021). Normalized preference score was also calculated as a CPP score percentage change from baseline to test [i.e., CPP % change = (preference % at Test—preference % at Baseline)/preference % at Baseline].

Locomotor data were also collected throughout each CPP session. Notably, the sample size for locomotor data does not match that of the CPP preference data because the CPP apparatus technically failed and stopped recording locomotion at random times for some sessions for different mice. For any mouse affected, the locomotor data were entirely removed because repeated-measures analyses require data from all sessions. The final sample sizes for locomotion analyses for saline groups were as follows: adolescent male *n* = 23, adolescent female: *n* = 16; adult male: *n* = 22; adult female *n* = 16 and for methamphetamine groups were as follows: adolescent male *n* = 17, adolescent female: *n* = 20; adult male: *n* = 20; adult female *n* = 19.

### Data Analyses

Statistical analyses were performed in SPSS Statistics 23 (IBM Corp., NY, United States), which showed that the present data were normally distributed. Chi-square test of independence analyzed CPP/CPA proportion data. The rest of the behavioral data were first analyzed with overall ANOVA with all the factors included to minimize type 1 error (McHugh, 2011). Between-subjects factors were as follows: Age (adolescents vs. adults), Sex (male vs. female), and Group (saline vs. methamphetamine in the afternoon sessions). Within-subjects factors were as follows: Day (baseline, conditioning days 1, 2, 3, and 4, or test), Time [locomotion at morning session (when all mice received saline) vs. afternoon session (when mice received either saline or methamphetamine, depending on their Group)], and Chamber [speed in morning chamber (saline-paired) vs. afternoon chamber (saline- or methamphetamine-paired)]. It should be noted that within-subjects factors depended on the measurements of interest for target outcomes analyzed in the results. Specifically, Time was only used to assess distance travelled for locomotor activity analyses during conditioning, while Chamber was only used to compare the speed in the two chambers at test.

Only significant interactions were followed up with *post hoc* ANOVAs (McHugh, 2011). Specifically, when Time (morning vs. afternoon session) significantly interacted with all other factors, we did *post hoc* ANOVAs examining interacting factors separated by the morning and afternoon sessions. When Group was involved in most of the interactions, the interacting factors were assessed with *post hoc* ANOVAs per Group. When Day was involved in more significant interactions than Group, *post hoc* ANOVAs examined the interacting factors separately for each Day. Alpha level (statistical significance) was determined at *p* ≤ 0.05, except for multiple group comparisons that were Bonferroni adjusted. Non-significant effects with *p* ≤ 0.1 were specified.

## Results

### Locomotor Activity

**FIGURE 2:**
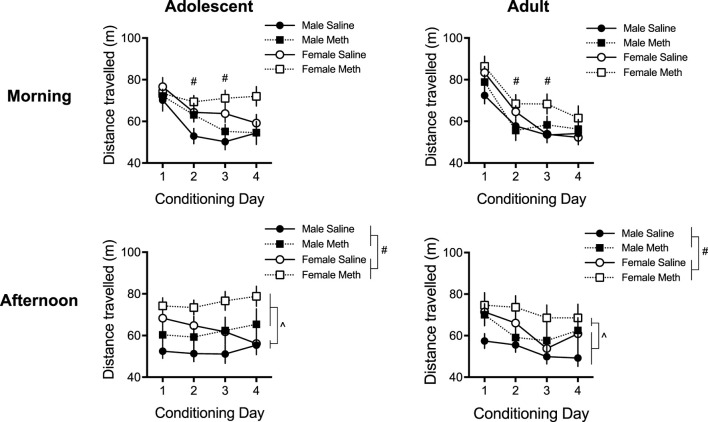
Total distance travelled (±SEM) in the morning and afternoon sessions following an injection of saline or methamphetamine (meth; 0.1 mg/kg) over 4 consecutive days (*n* = 16–23 per group). Note that all mice received saline in the morning sessions. Effect of Sex: (#) *p* < 0.05. Effect of Group: (˄) *p* < 0.05.

A five-way repeated measures ANOVA of the distance travelled during each conditioning session revealed significant effects of Day [F(3,435) = 43.0; *p* < 0.0001], Sex [F(1,145) = 13.7; *p* < 0.0001] and Group [F(1,145) = 8.5; *p* = 0.004] (Figure 2). There was also a significant Time × Day × Sex × Age interaction [F(3,435) = 3.4; *p* = 0.017] [no other four- or five-way interactions were observed (smallest *p* = 0.16)]. *Post hoc* ANOVAs per Time were conducted as described in the methods to investigate the distance travelled in the morning sessions (saline) separately from the afternoon sessions (saline or methamphetamine), which are described below.

A four-way repeated measures ANOVA of the morning Time locomotion showed significant effects of Day [F(3,435) = 79.6; *p* < 0.0001] and Sex [F(1,145) = 9.9; *p* = 0.002], and significant interactions of Day × Age [F(3, 435) = 6.0; *p* = 0.001] and Day × Sex × Age [F(3,435) = 3.9; *p* = 0.009]. No other effects/interactions were detected (smallest *p* = 0.060, Day × Sex × Group). To understand this Day × Sex × Age interaction, we analyzed Sex and Age group effects in each Day (Bonferroni adjusted *p* < 0.013 of significance, 0.05/4 to account for four multiple comparisons). This showed that females moved more than males on days 2 and 3 {effect of Sex in the second [F(1,149) = 12.4; *p* = 0.001] and third [F(1,149) = 12.8; *p* < 0.0001] day}, suggesting that females habituated to morning saline injections slower than males. By the fourth morning saline injection they travelled a distance similar to males. No effects of Age passed Bonferroni corrections for any day in the morning session. No Sex × Age interaction was detected in any morning session (smallest *p* = 0.14).

A similar analysis of the afternoon Time locomotion yielded significant effects of Sex [F(1,145) = 13.7; *p* < 0.0001] and Group [F(1,145) = 11.6; *p* = 0.001], indicating that females moved more than males overall, and methamphetamine groups moved more than saline groups overall. There was also a significant effect of Day [F(3,435) = 4.7; *p* = 0.003] and a significant Day × Age interaction [F(3,435) = 4.1; *p* = 0.007]. No other effects/interactions were observed (smallest *p* = 0.10, Day × Group). We analyzed Age effects in each Day to understand the Day × Age interaction (Bonferroni adjusted *p* < 0.013 of significance, 0.05/4 to account for four multiple comparisons), which revealed no significant Age effects on any Day (smallest *p* = 0.10, Day 4).

### Conditioned Hyperactivity

**FIGURE 3:**
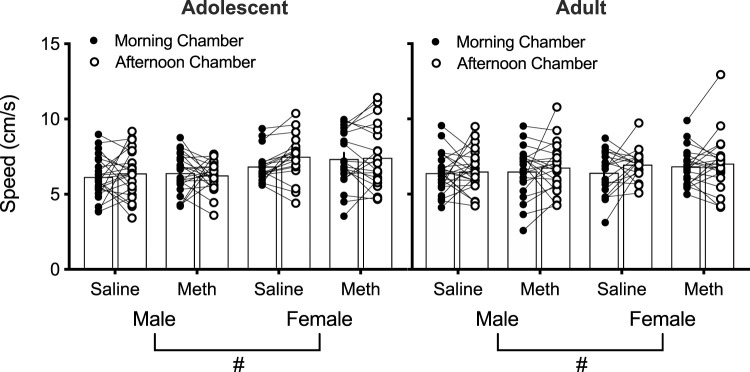
Individual speed (cm/s) in the morning and afternoon chambers at test in saline-treated and methamphetamine (meth)-treated mice (*n* = 18–24 per group) as a measure of conditioned hyperactivity. Effect of Sex: (#) *p* < 0.05.

A four-way repeated measures ANOVA of the speed (cm/s) at test in the morning vs. afternoon chambers as a measure of conditioned hyperactivity revealed a significant effect of Sex [F(1,158) = 9.8; *p* = 0.002] (Figure 3). No other effects/interactions were observed (smallest *p* = 0.051, Chamber). These results suggest that saline or 0.1 mg/kg methamphetamine injections do not cause conditioned hyperactivity and females move faster than males overall in mice.

### Conditioned Place Preference

**FIGURE 4:**
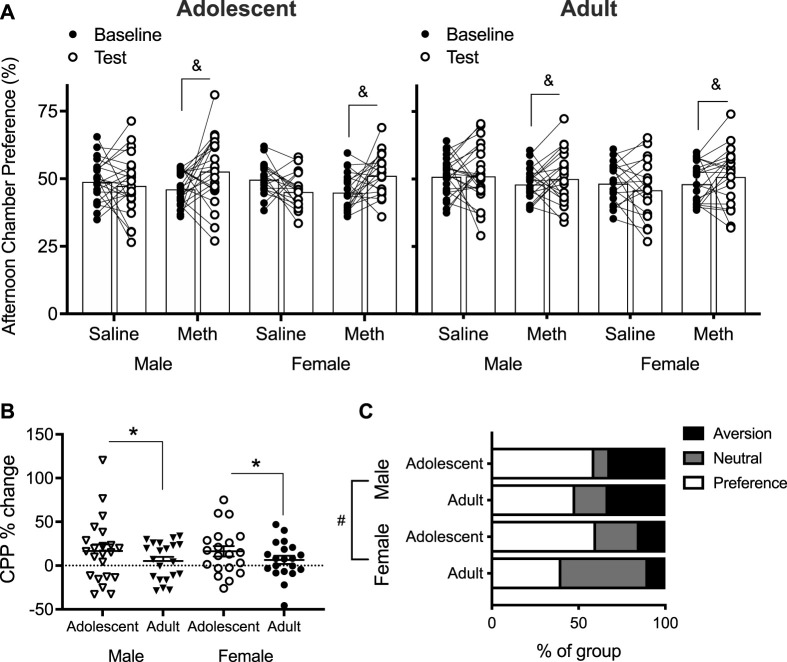
**(A)** Conditioned place preference (CPP) % formed by saline-treated and methamphetamine (meth)-treated mice at baseline and test (*n* = 18–24 per group). Percent preference was calculated by dividing time spent in the afternoon chamber by the combined time spent in both chambers. Columns show mean (±SEM). Effect of Day only in meth group: (&) *p* < 0.05. **(B)** CPP % change at test normalized to baseline in meth-treated mice only [i.e., CPP % change = (% at test–% at baseline)/% at baseline]. Effect of Age: (*) *p* < 0.05. **(C)** Based on normalized CPP % change, proportion of mice that formed a preference (CPP % change > 10%), remained neutral (CPP % change between 10% and −10%), and formed an aversion (CPP % change < −10%) to the meth (0.1 mg/kg)-paired chamber at test. Effect of Sex: (#) *p* < 0.05.

A four-way repeated measures ANOVA across baseline and test % preference for the afternoon chamber showed a significant Day × Group interaction [F(1,158) = 12.9; *p* < 0.0001] (Figure 4A). No other effects/interactions were observed (smallest *p* = 0.098, Day × Age × Group). When the effect of Day was assessed as *post hoc* ANOVA in each Group to understand this Day × Group interaction, Day had no effect (*p* = 0.12) in saline groups. In the methamphetamine groups, however, there was a significant effect of Day [F(1,82) = 11.4; *p* = 0.001]. This suggests that at the group level, mice treated with 0.1 mg/kg methamphetamine formed CPP after 4 days of conditioning, independently of Age and Sex.

Individually normalized CPP % change from baseline to test was analyzed in the methamphetamine groups only (Figure 4B), based on the evidence that saline groups did not form CPP (Figure 4A). Two-way ANOVA revealed a significant effect of Age [F(1,79) = 7.4; *p* = 0.008], with adolescents showing a greater change than adults. No effect of Sex [F(1,79) = 1.1; *p* = 0.30] nor Age × Sex interaction [F(1,790 = 0.7; *p* = 0.39] were detected. Taken together, adolescents appear to form a stronger preference to 0.1 mg/kg methamphetamine than adults in male and female mice.

Importantly, normalized data show a clear evidence of CPA, albeit only in a few mice (Figure 4B). Therefore, we compared groups on the proportion of methamphetamine-treated mice that formed a preference (defined as change in preference >10%), remained neutral (defined as change in preference between 10% and −10%), and formed an aversion (defined as change in preference < −10%) to the methamphetamine-paired chamber. These numbers are based on previously published studies that frequently report 10% change for CPP and CPA [see Cullity et al. (2021) for more detail]. Chi-square test of independence showed a significant effect of Sex [χ^2^(2) = 8.2; *p* = 0.016] but no effect of Age [χ^2^(2) = 3.5; *p* = 0.20]. Inspection of data shows that this result is driven by fewer females forming an aversion compared to males (Figure 4C).

## Discussion

We investigated age and sex differences in CPP and locomotor activity to 0.1 mg/kg methamphetamine in mice. As a group, mice developed CPP for the methamphetamine-paired chamber after 4 days independently of age or sex. However, baseline-normalized scores indicated that adolescents formed a stronger preference compared to adult mice, and females were less likely to form CPA compared to males. Females also habituated to saline injections more slowly than males. While mice moved more after an injection of 0.1 mg/kg methamphetamine compared to saline, this dose did not produce conditioned hyperactivity.

### Adolescents Form Stronger Preference to 0.1 mg/kg Methamphetamine Than Adults

In the present study, adolescents formed a stronger preference compared to adult mice when normalized changes in CPP were analyzed (Figure 4B). We previously reported no differences in normalized preference when mice were conditioned to a high dose (3 mg/kg) of methamphetamine (Cullity et al., 2021). Together, these results strongly suggest that adolescent mice are hypersensitive to the rewarding properties of low (e.g., 0.1 mg/kg) but not high (e.g., 3 mg/kg) dose methamphetamine, as detected by CPP. Using different doses of the same drug to examine potential CPP and CPA is helpful to assess rewarding and aversive effects of a drug (Tzschentke, 2014). Importantly, the present study's 0.1 mg/kg dose of methamphetamine produced a higher CPP in adolescents compared to adolescents in the previous study using 3 mg/kg (Cullity et al., 2021). Opposite results are observed for adults, with 0.1 mg/kg inducing lower CPP than 3 mg/kg (Cullity et al., 2021). These findings indicate that the dose–reward response curve may be left-shifted in adolescents compared to adults for methamphetamine. In contrast, the proportion of mice that show CPA does not appear to be different between the two different doses used in the present study and a previous study (Cullity et al., 2021). Based on these observations that dose changes affected CPP but not CPA, we propose that at least for methamphetamine, feelings of reward and aversion are separate and unrelated processes. This differs from nicotine, which appears to increasingly induce CPA with dose increase suggesting that the same rewarding process becomes aversive with dose escalation (Le Foll and Goldberg, 2005).

Hypersensitivity to rewarding effects of 0.1 mg/kg methamphetamine observed in adolescents does not appear to generalize to reinforcing effects of methamphetamine tested by self-administration studies. For example, adolescents do not self-administer more methamphetamine than adults in typical 2 h daily sessions (at 0.03 or 0.05 mg/kg/infusions) (Anker et al., 2012; Luikinga et al., 2019), although adolescents do self-administer more than adults when given access to methamphetamine for an extended period of time (6 h sessions) (Anker et al., 2012). These findings indicate that conditions to detect rewarding vs. reinforcing properties of methamphetamine significantly differ, with adolescents' hypersensitivity to reward affected by dose, and hypersensitivity to reinforcing properties affected by availability and access. Interestingly, the present findings show Pavlovian conditioning to the context using methamphetamine differs between adolescents and adults, which suggests that context-driven reinforcement learning [i.e., self-administration without discrete cue (Kim et al., 2015)] may also be affected by age.

### Sex-Dependent Aversion to 0.1 mg/kg Methamphetamine

We observed that female mice were less likely to form an *aversion* to 0.1 mg/kg methamphetamine compared to males, regardless of age (Figure 4C). In contrast, Chen et al. (2003) reported that adult female mice were more likely to develop CPP to a moderate dose of methamphetamine (1 mg/kg) compared to adult males at group level (Chen et al., 2003). Our results suggest that a reduced propensity to form aversion in females compared to males may have contributed towards sex differences observed in that study. Importantly, these findings are unlikely to be caused by sex differences in methamphetamine metabolism, considering that urinary excretion of methamphetamine and its metabolites do not differ between male and female rodents (Yamada et al., 1986). The reduced susceptibility of females to the aversive effects of methamphetamine compared to males may not be dose sensitive, being observed previously with 3 mg/kg of methamphetamine (Cullity et al., 2021). This is in contrast with a two-choice bottle study, which reported an *increase* in taste aversion with increasing amphetamine doses in male and female rats (Infurna and Spear, 1979). Future studies should aim to further characterize the dose–response relationship between methamphetamine and its aversive effects in females.

In humans, females tend to transition more rapidly from experimental to regular and problematic methamphetamine use (Brecht et al., 2004; Mayo et al., 2019). In addition, adolescent females tend to use methamphetamine at higher rates than males of the same age (Rawson et al., 2005) and are more likely to report methamphetamine dependence (Kim and Fendrich, 2002; Dluzen and Liu, 2008). Moreover, evidence suggests that men and women use a similar dose of methamphetamine, even after controlling for age and body mass index (He et al., 2013). In light of those findings, our results suggest that females may be less likely to experience the negative effects of methamphetamine, such as aggression or anxiety, and thus more likely to engage in sustained use after initiation. Future studies assessing sex differences in the negative effects of methamphetamine in humans appears necessary to understand female vulnerability.

### Methamphetamine-Induced Hyperactivity to 0.1 mg/kg Methamphetamine Is Sex- but Not Age-Dependent

In the second and third morning sessions, females were more hyperactive compared to males when exposed to saline. These differences were not observed in our previous study (Cullity et al., 2021). Such slowed habituation may be related to clear sex differences during the afternoon session in which females moved more than males each day. Importantly, there were no sex differences on the fourth morning session, suggesting that female mice reached the same level of habituation to the morning saline injections as male mice.

In the afternoons, methamphetamine groups moved more than the saline groups overall. This is in contrast with a previous study reporting no differences between saline- and methamphetamine-treated male rats at low dose (0.125 mg/kg) (Zakharova et al., 2009). Sessions in that study were 1 h in length, compared to 30 min in the present study. It may be that animals treated with a low dose of methamphetamine move more than saline animals at the start of the session before reaching a plateau over time, which may reduce the detection of overall effects. In addition, methamphetamine may have a strain-specific motor effect (Good and Radcliffe, 2011). Therefore, it may be that 0.1 mg/kg methamphetamine is sufficient to induce locomotor hyperactivity in Swiss mice, whereas 0.125 mg/kg is too low to induce hyperactivity in rats.

Notably, we did not find any age-dependent locomotor effect. Previous studies have reported that male adult rats travel more than adolescent when exposed to 0.5 mg/kg methamphetamine (Zakharova et al., 2009). In male mice, adults displayed heightened locomotion compared to adolescents to 2 mg/kg, but not 4 mg/kg of methamphetamine (Zombeck et al., 2008). In contrast, we previously showed that adolescent mice moved *more* than adults in response to 3 mg/kg methamphetamine in the last day of conditioning (Cullity et al., 2021). Taken together, this suggests that adolescents may be more sensitive to the dose changes of methamphetamine in affecting locomotion, with hyperlocomotion and sensitization emerging at a higher dose.

### Mice Do Not Display Conditioned Hyperactivity to 0.1 mg/kg Methamphetamine

In the present study, we did not observe conditioned hyperactivity to 0.1 mg/kg methamphetamine, regardless of age or sex. While most previous studies assessing CPP to low doses of methamphetamine did not report conditioned hyperactivity data (Schindler et al., 2002; Zakharova et al., 2009; Su et al., 2013), our findings are consistent with a study in adult male rats reporting no conditioned hyperactivity after conditioning with low doses of methamphetamine (0.0625 or 0.125 mg/kg) (Bevins and Peterson, 2004). Results in the present study therefore extend this finding to adolescent and adult mice of both sexes.

Previous studies assessing conditioned hyperactivity at moderate doses of methamphetamine (0.25–2 mg/kg) did not include rigorous control groups to assess whether hyperactivity in the methamphetamine chamber was in fact related to conditioning (Bevins and Peterson, 2004; Chesworth et al., 2013; Kim et al., 2013). For example, while adult males were reported to display conditioned hyperactivity to 0.25, 0.5, and 1 mg/kg methamphetamine, the saline-paired control chamber was not assessed (Bevins and Peterson, 2004). It is therefore possible that increased activity may simply be because of repeated methamphetamine injections in any chamber rather than conditioned pairing with the chamber. Likewise, we previously reported that when exposed to 2 mg/kg methamphetamine, adult male mice move faster in the methamphetamine-paired chamber at test compared to adolescents (Kim et al., 2013), an effect that may not be specific to the methamphetamine-paired chamber because activity in the saline-paired chamber at test was not analyzed. Another study using 2 mg/kg methamphetamine reported adult male mice do not display conditioned hyperactivity (Chesworth et al., 2013). That study, however, analyzed the distance travelled in the saline- and methamphetamine-paired chambers combined at habituation and at test and did not include a saline-only comparison group (Chesworth et al., 2013). Any potential hyperactivity may therefore not be associated with the methamphetamine-paired context over control context and is not suitable to study Pavlovian conditioning-based mechanisms of addiction. This is notable because Pavlovian conditioning plays a critical role in response to methamphetamine-related cues in humans (Guerin et al., 2021a), which may lead to relapse after abstinence (Carter and Tiffany, 1999). Our results indicate that at least for methamphetamine, hyperactivity does not appear to be a conditioned response to context with methamphetamine experience.

### Limitations, Future Directions, and Conclusion

Age and sex differences to moderate doses of methamphetamine remain to be investigated for a comprehensive understanding of how methamphetamine is processed as a reward in different demographics. In addition, previous studies in other psychostimulants have reported age differences in extinction and reinstatement of place preference (Brenhouse and Andersen, 2008; Guerin et al., 2021b). Relapse after prolonged abstinence is also an important feature in addiction. Future studies should therefore investigate age and sex differences in extinction, abstinence, relapse, and reinstatement of methamphetamine CPP.

Dopamine receptors appear to play a key role in place preference (Hoffman and Beninger, 1988; White et al., 1991). Dopamine receptor 1 and 2 expression changes dramatically across adolescence in male and female mice in several brain regions including mesocorticolimbic dopaminergic regions (Andersen et al., 2000; Kim et al., 2017; Cullity et al., 2019), and such changes have been proposed to underlie adolescent vulnerability to mental disorders, including addiction (Andersen et al., 2000; Spear, 2000; Caballero et al., 2016; Zbukvic and Hyun Kim, 2018). It would therefore be informative to assess dopamine receptor expression in addiction-relevant regions such as the nucleus accumbens and the prefrontal cortex in response to this low dose of methamphetamine that leads to age and sex differences in CPP.

The present and the previous study (Cullity et al., 2021) in separate mice assessed a control condition in which all mice received saline in morning and afternoon chambers. We feel scientifically compelled to highlight that individual data clearly show how most of the mice receiving saline changed their preference from baseline to test. That is, if they were below 50% at baseline, they went above 50% at test, and vice versa. Because we chose an unbiased allocation (starting preference is ∼50% at the group level), these changes cancelled each other out, resulting in no evidence of CPP or CPA at the group level in saline mice. However, such findings consistently observed across >100 mice in two studies raise a disturbing possibility that biased allocation of the baseline chamber (i.e., less-preferred or more-preferred side to observe CPP or CPA, respectively) can artificially induce CPP or CPA and does not indicate of rewarding or aversive experience from the drug. Our findings strongly suggest that rodents like to explore at test the chamber they spent less time in during baseline. To avoid misinterpretation of changing preference as drug-based, unbiased allocation is strongly recommended for future CPA and CPP studies.

In conclusion, the present study observed that adolescent mice formed a stronger preference to the low dose of methamphetamine compared to adults. In addition, females were less likely to form an aversion to methamphetamine compared to males at an individual level. These results suggest that adolescents are more sensitive to the rewarding effects of methamphetamine compared to adults, which may, at least in part, drive the heightened vulnerability to substance use disorders in youths. Female resistance to the aversive effects of methamphetamine may explain how, in humans, females transition to regular methamphetamine use more quickly than males.

## Data Availability

Data are available upon request to the corresponding author.
